# IMPACT OF A POLICY CHANGE ON ADULT PROTECTIVE SERVICES CASE VOLUME AND SERVICES IN CALIFORNIA

**DOI:** 10.1093/geroni/igae098.2927

**Published:** 2024-12-31

**Authors:** Zachary Gassoumis, Erin Thayer, Jeanine Yonashiro-Cho

**Affiliations:** University of Southern California, Los Angeles, California, United States; Keck School of Medicine of USC, University of Southern California, Department of Family Medicine, Keck School of Medicine of USC, University of Southern California, California, United States; University of Southern California Keck School of Medicine, Los Angeles, California, United States

## Abstract

The adult protective services (APS) system in the U.S. investigates and provides services to older adults and younger adults with a disability or vulnerability who are experiencing abuse, neglect, self-neglect, or financial exploitation. APS systems are undergoing shifts in their scope and nature of services, due to national movements toward program standardization, population aging, and changing population dynamics. California recently implemented a policy change, effective January 2022, that expanded APS services to all adults age 60 and older (reduced from 65) and expanded self-neglect to include individuals experiencing homelessness. We analyzed publicly available APS records from October 2019 through October 2023, using segmented linear regression on month-level data to assess the impact of this policy on overall APS case volume, number of APS clients aged 60-64, and clients referred to or receiving housing services. Following the January 2022 implementation, there was an increase in the growth of overall APS cases over time (b=103.9, p=.015) but no significant increase in the level. Among clients aged 60-64, there was a significant increase in both level and growth of cases over time, jumping from roughly 375 to 550 monthly cases immediately following the policy implementation and growing to 800 cases by October 2023. Similar results were seen for housing services, with significant increases in the growth of cases over time (b=10.2, p=.005). This growth in volume and service type has implications for programmatic refinements and for better understanding resource needs in the light of national discussions on APS standardization and scope of services.

